# Complete Genome Sequence of Elephant Endotheliotropic Herpesvirus 1A

**DOI:** 10.1128/genomeA.00106-13

**Published:** 2013-04-11

**Authors:** Paul D. Ling, Jeffrey G. Reid, Xiang Qin, Donna M. Muzny, Richard Gibbs, Joseph Petrosino, RongSheng Peng, Jian-Chao Zong, Sarah Y. Heaggans, Gary S. Hayward

**Affiliations:** Department of Molecular Virology and Microbiology, Baylor College of Medicine, Houston, Texas, USAa;; Human Genome Sequencing Center, Baylor College of Medicine, Houston, Texas, USAb;; Viral Oncology Program, Johns Hopkins School of Medicine, Baltimore, Maryland, USAc

## Abstract

Elephant endotheliotropic herpesvirus 1A is a member of the *Proboscivirus* genus and is a major cause of fatal hemorrhagic disease in endangered juvenile Asian elephants worldwide. Here, we report the first complete genome sequence from this genus, obtained directly from necropsy DNA, in which 60 of the 115 predicted genes are not found in any known herpesvirus.

## GENOME ANNOUNCEMENT

The *Proboscivirus* genus was tentatively assigned within the *Betaherpesvirinae* subfamily (1), but PCR analysis of conserved genes in seven distinct elephant endotheliotropic herpesvirus (EEHV) species from both Asian and African elephant hosts (2, 3) has led us to propose (4) that they should instead be considered a new *Deltaherpesvirinae* subfamily of mammalian herpesviruses. EEHVs cannot yet be propagated in cell culture, and the only source of viral genomic DNA available has been from necropsy tissue or viremic blood samples. Both walking procedures (5) and partial phage lambda libraries (6) have been used to characterize up to 85 kb of the central conserved core region of several strains, including the partially chimeric variant EEHV1B, but numerous additional novel genes were expected. Therefore, we used a next-generation approach to determine the complete sequence of EEHV1A from a 12-year-old hemorrhagic disease case named “Kimba.”

Twenty-two gigabases of raw sequence data reads from two paired-end Illumina runs were assembled *de novo* and confirmed and corrected by extensive targeted local PCR sequencing. The sequenced necropsy DNA sample was a mixture of EEHV DNA with host *Elephas maximus* DNA, neither of which had a close reference sequence available. However, a subset of expected viral reads with copy number between 25- and 40-fold relative to a single-copy host gene were selected for assembly by the program Velvet, yielding 417 contigs, including one of 83.7 kb. A draft final assembly was generated by (i) joining the six largest contigs totaling 162 kb by PCR amplification across adjacent ends, (ii) manual elimination of all contigs with features resembling host cell repetitive DNA, and (iii) successful amplification of a 15.7-kb PCR product that physically joined across the left and right outside ends. The eleven remaining contigs from the manual elimination step additively matched exactly to the PCR product in the amplification step; all were confirmed to be viral by PCR amplification from a set of six standard strains of EEHV-positive *Elephas* DNA, but not from three EEHV-negative *Elephas* DNA samples.

The complete 177,136-bp EEHV1A (Kimba) genome consists of 115 identified likely open reading frames (ORFs), including 14 for which we were able to assign likely splicing patterns. The protein-coding genes can be categorized into 37 conserved core genes common to all herpesviruses (all >50% diverged from their nearest orthologs), plus 15 genes in common with either both betaherpesviruses and gammaherpesviruses or just betaherpesviruses, three other core genes that are missing in human cytomegalovirus (HCMV) (OBP, RRB, and TK), and 60 novel ORFs not found in any other herpesviruses. The last group includes captured cellular genes vGCNT1, vFUT9, three versions of vOX2, five other immunoglobulin family proteins, and a large cluster of tandemly repeated highly diverged 7× transmembrane (TM) proteins, some of which resemble viral G-protein-coupled receptors (vGPCRs) and chemokine receptors. Furthermore, a 40-kb core segment of the *Proboscivirus* genome is inverted relative to the organization in *Betaherpesvirinae*. Phylogenetic trees of the core proteins are consistent with the designation of the probosciviruses as a new subfamily branching between the *Betaherpesvirinae* and *Gammaherpesvirinae*.

### Nucleotide sequence accession number. 

The genome sequence of EEHV1A (Kimba) has been deposited in GenBank under the accession no. KC618527.

## References

[1] Davison AJ Eberle R Ehlers B Hayward GS McGeoch DJ Minson AC Pellett PE Roizman B Studdert MJ Thiry E 2009 The order *Herpesvirales*. Arch. Virol. 154:171–177 19066710 10.1007/s00705-008-0278-4 PMC3552636

[2] Latimer E Zong JC Heaggans SY Richman LK Hayward GS 2011 Detection and evaluation of novel herpesviruses in routine and pathological samples from Asian and African elephants: identification of two new probosciviruses (EEHV5 and EEHV6) and two new gammaherpesviruses (EGHV3B and EGHV5). Vet. Microbiol. 147:28–41 20579821 10.1016/j.vetmic.2010.05.042 PMC2976818

[3] Richman LK Montali RJ Garber RL Kennedy MA Lehnhardt J Hildebrandt T Schmitt D Hardy D Alcendor DJ Hayward GS 1999 Novel endotheliotropic herpesviruses fatal for Asian and African elephants. Science 283:1171–1176 10024244 10.1126/science.283.5405.1171

[4] Hayward GS 2013 Multiple new variants and sub-types of elephant gammaherpesviruses (EGHVs) and deltaherpesviruses (EEHVs). Ninth Annual International Elephant Endotheliotropic Herpes Virus (EEHV) Workshop, Houston, TX.

[5] Ehlers B Dural G Marschall M Schregel V Goltz M Hentschke J 2006 Endotheliotropic elephant herpesvirus, the first betaherpesvirus with a thymidine kinase gene. J. Gen. Virol. 87:2781–2789 16963736 10.1099/vir.0.81977-0

[6] Richman LK 2003 Pathological and molecular aspects of fatal endotheliotropic herpesviruses. Ph.D. thesis. Johns Hopkins University, Baltimore, MD

